# Molecular and Functional Imaging of Internet Addiction

**DOI:** 10.1155/2015/378675

**Published:** 2015-03-24

**Authors:** Yunqi Zhu, Hong Zhang, Mei Tian

**Affiliations:** ^1^Department of Nuclear Medicine, The Second Hospital of Zhejiang University School of Medicine, 88 Jiefang Road, Hangzhou, Zhejiang 310009, China; ^2^Zhejiang University Medical PET Center, Hangzhou 310009, China; ^3^Institute of Nuclear Medicine and Molecular Imaging, Zhejiang University, Hangzhou 310009, China; ^4^Key Laboratory of Medical Molecular Imaging of Zhejiang Province, Hangzhou 310009, China

## Abstract

Maladaptive use of the Internet results in Internet addiction (IA), which is associated with various negative consequences. Molecular and functional imaging techniques have been increasingly used for analysis of neurobiological changes and neurochemical correlates of IA. This review summarizes molecular and functional imaging findings on neurobiological mechanisms of IA, focusing on magnetic resonance imaging (MRI) and nuclear imaging modalities including positron emission tomography (PET) and single photon emission computed tomography (SPECT). MRI studies demonstrate that structural changes in frontal cortex are associated with functional abnormalities in Internet addicted subjects. Nuclear imaging findings indicate that IA is associated with dysfunction of the brain dopaminergic systems. Abnormal dopamine regulation of the prefrontal cortex (PFC) could underlie the enhanced motivational value and uncontrolled behavior over Internet overuse in addicted subjects. Further investigations are needed to determine specific changes in the Internet addictive brain, as well as their implications for behavior and cognition.

## 1. Introduction

Addiction to substances or activities can profoundly affect people's health and sometimes lead to serious social problems [1–3]. For example, maladaptive use of the Internet can result in the development of a behavioral addiction, leading to significantly clinical impairment or distress [4]. Recently, research about Internet addiction (IA), especially Internet gaming disorder (IGD), has increased both in quantity and in quality [5, 6]. IA is usually defined as an inability of individuals to control their Internet use, resulting in marked psychological, social, and/or work difficulties [7]. IA is associated with various negative consequences, such as sacrificing real-life activities, lack of attention, aggression and hostility, stress, dysfunctional coping, worse academic achievement, low well-being, and high loneliness [5].

While IA has drawn growing attention from scientific world, there are currently no standard diagnostic criteria. Several diagnostic criteria have been proposed to quantify IA. The most widely used diagnostic criterion is Young's Diagnostic Questionnaire [8–10]. Based on the Diagnostic and Statistical Manual of Mental Disorders (DSM-IV), Young initially developed a short eight-item questionnaire that assessed IA [8]. In employing these criteria, participants with five or more of the eight criteria presented during the past 6 months were classified as suffering from IA. Young also created a 20-item questionnaire, called the Internet Addiction Test [10]. In the 20-item questionnaire, each item is based on a 5-point Likert scale evaluating the degree of problems caused by Internet use. Scores over 50 indicate occasional or frequent internet related problems and scores over 80 indicate significant IA-related life problems [10]. The Internet Addiction Test was proved to be a valid and reliable instrument that can be used in classifying IA [11]. Other diagnostic criteria and screening instruments have also been created and used to assess IA [12–16].

As an important subtype of IA, IGD has gained more and more attention from the whole world. IGD has been included in the appendix of the DSM-V, with a goal of encouraging additional studies [4]. The DSM-V describes IGD as a “persistent and recurrent use of the Internet to engage in games, often with other players, leading to clinically significant impairment or distress as indicated by five or more (criteria) in a 12-month period” [5].

In the past few years, molecular and functional imaging techniques have been increasingly used to study the neurobiological mechanism underlying IA. Molecular imaging is a rapidly developing field aimed to provide disease-specific molecular information through diagnostic imaging studies [17]. The term molecular imaging can be broadly defined as the in vivo characterization and measurement of biologic processes at the cellular and molecular level [18]. In order to prevent and treat IA, it is important to have a clear understanding of its underlying mechanisms. Technological advances have led to great use of both structural and functional brain imaging modalities, for example, magnetic resonance imaging (MRI), positron emission tomography (PET), and single photon emission computed tomography (SPECT), to assist with the diagnosis of different clinical diseases as well as the study of IA. Here we review recent molecular and functional imaging studies that have provided considerable insight into the neurobiological mechanisms of IA, focusing particularly on MRI and PET imaging approaches.

## 2. MRI Findings

MRI is a highly versatile imaging modality which uses magnet and radiofrequency energy to visualize the internal structure and soft tissue morphology of the body [19]. The primary advantage of MRI as a molecular imaging modality is its high spatial resolution (micrometers), which allows physiological and anatomical information to be extracted simultaneously. Functional MRI (fMRI) is a noninvasive technique which can be used to monitor metabolic activity changes in brain [20]. It has been verified that an increase in neuronal activity within a certain brain region leads to a net increase in the amount of oxygenated blood flow in that specific region [21]. Since deoxygenated hemoglobin is paramagnetic, and oxygenated hemoglobin is diamagnetic, the blood-oxygen-level-dependent (BOLD) contrast enables the examination of regional brain functioning across different contexts and cognitive demands.

### 2.1. Structural Changes

**Figure 1:**
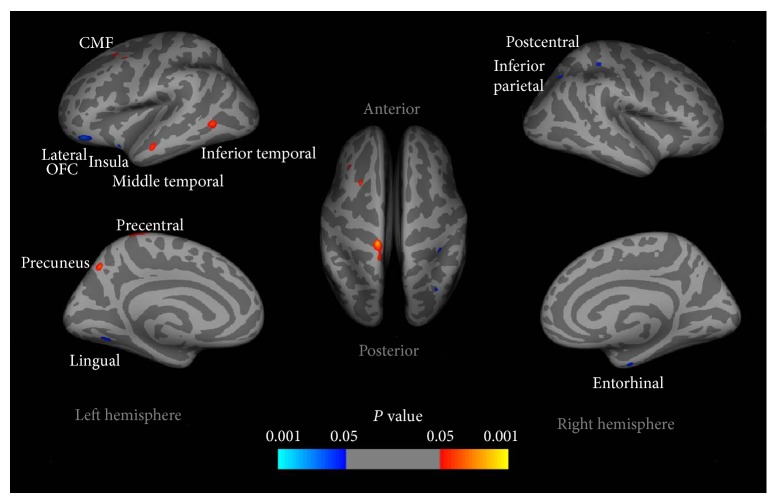
Cortical thickness differences in adolescents with IGD compared with healthy controls. Increased cortical thickness was observed in several regions in adolescents with IGD compared to healthy controls, that is, the left precentral cortex, precuneus, middle frontal cortex, and inferior temporal and middle temporal cortices. Reduced cortical thickness in the left lateral OFC, insula cortex, and lingual gyrus, along with the right postcentral gyrus, entorhinal cortex, and inferior parietal cortex were detected in adolescents with IGD [23].

Using MRI, some studies have shown that brain structural changes are associated with IA. Using the Stroop color-word test [22], which has been widely used for assessing inhibitory control, a study reported that adolescents with IGD showed impaired cognitive control ability [23]. Imaging results demonstrated that brain regions associated with executive function, for example, the left lateral orbitofrontal cortex (OFC), insula cortex, and entorhinal cortex, showed decreased cortical thickness in IGD subjects compared with controls (Figure 1). Moreover, the authors also reported that the reduced cortical thickness of the left lateral OFC was correlated with the impaired cognitive control ability in IGD adolescents. Consistent with this, another study also reported reduced thickness in the OFC of Internet addicted adolescents [24]. Given the view that the OFC is implicated in the pathology of drug and behavioral addictions [25, 26], the authors suggest that IA shares similar neurobiological mechanism with other addictions. Apart from the decreased cortical thickness, increased cortical thickness was also observed in the left precentral cortex, precuneus, middle frontal cortex, and inferior temporal and middle temporal cortices [23] (Figure 1). The precuneus is associated with visual imagery, attention, and memory retrievals [27]. The inferior temporal cortex and the middle frontal cortex have been shown to engage in craving induced by drug cues [28, 29]. Therefore, these results suggest that the increased cortical thickness areas in IGD may be associated with craving of gaming cues.

Voxel-based morphometry is an unbiased technique for characterizing regional cerebral volume and tissue concentration differences in structural magnetic resonance images [30, 31]. Voxel-based morphometry has been useful in identifying subtle structural abnormalities in a variety of neurological diseases. Voxel-based morphometry studies demonstrated that IGD adolescents had lower grey matter density in the left anterior cingulate cortex (ACC), left posterior cingulate cortex (PCC), left insula, and left lingual gyrus [32]. Using the same technique, decreased gray matter volume was found in the bilateral dorsolateral PFC, supplementary motor area, OFC, cerebellum, and left rostral ACC in another group of Internet addicted adolescents [33]. Additionally, a third Voxel-based morphometry study reported gray matter atrophy in the right OFC, bilateral insula, and right supplementary motor area of IGD [34]. The results of gray matter atrophy among these studies were not consistent, which may be due to different data processing methods. The PFC has been implicated in planning complex cognitive behavior, personality expression, and decision making, which consists of the dorsolateral PFC, ACC, and OFC [35]. Numerous imaging studies have brought to light the role of the PFC in addiction [36]. Now it is commonly recognized that the OFC plays a key role in impulse control and decision making [26, 37]. Functional brain imaging studies have revealed that the dorsolateral PFC and rostral ACC were involved in cognitive control [38, 39]. Reduced gray matter volume in the PFC may be associated with uncontrolled behavior in Internet addicts, which may explain fundamental symptoms of IA. The insula has been proposed to play a crucial role in addiction [40]. A number of functional imaging studies provide evidence that the insula is necessary for the explicit motivation to take drugs, and this function is common among drug abusers [41, 42]. Therefore, these results are in agreement with previous findings and verified the necessary role of the PFC and insula for addiction.

Diffusion tensor imaging (DTI) is an approach available to track brain white matter fibers noninvasively. Water molecules' diffusion was found to be much faster along the white matter fibers than perpendicular to them. The difference between these two motions is the basis of DTI [43, 44]. DTI provides a framework for acquisition, analysis, and quantification of the diffusion properties of white matter. In addition to gray matter abnormalities, white matter abnormalities have also been suggested in IGD. Using DTI, a study assessed white matter integrity in individuals with IGD [45]. Higher fractional anisotropy was reported in the thalamus and left PCC in IGD relative to healthy controls. Moreover, higher fractional anisotropy in the thalamus was associated with greater severity of IGD. White matter abnormalities were also reported in other brain regions by other studies. For example, both enhanced and reduced fractional anisotropy were reported in a study, with enhanced fractional anisotropy in the left posterior limb of the internal capsule and reduced fractional anisotropy in the right parahippocampal gyrus [33]. In another study, significantly lower fractional anisotropy was reported throughout Internet addicts' brain, including the PFC and ACC [46]. However, no areas of higher fractional anisotropy were found. Similar results were also reported in another group of adolescents with IGD [34]. These findings suggest that IA disorder exhibit widespread white matter abnormalities, which may be linked to some behavioral impairment. It should be noted that the fractional anisotropy alterations in brain areas are not consistent in these studies, and the inconsistency in these studies needs further investigation.

### 2.2. Functional Abnormalities

Using arterial spin-labeling perfusion fMRI, Feng et al. investigated the effects of IGD on resting cerebral blood flow in adolescents [47]. Compared with control subjects, adolescents with IGD showed significantly higher global cerebral blood flow in the left inferior temporal lobe/fusiform gyrus, left parahippocampal gyrus/amygdala, right medial frontal lobe/ACC, left insula, right insula, right middle temporal gyrus, right precentral gyrus, left supplementary motor area, left cingulate gyrus, and right inferior parietal lobe. Most of these areas were included in a model proposed by Volkow et al. in which addiction emerges as an imbalance in information processing and integration among various brain circuits and functions [48]. Among these brain areas, the amygdala and hippocampus are part of a circuit involved in learning and memory that has been associated with craving in response to drug-associated cues [49]. Both the insula and the PFC are known to play a crucial role in addiction [36, 40]. Decreased cerebral blood flow was found in the left middle temporal gyrus, left middle occipital gyrus, and right cingulate gyrus in IGD adolescents. The results demonstrate that IGD alters cerebral blood flow distribution in adolescents' brain. However, it is unclear whether these cerebral blood flow alterations reflected primarily neurological lesions or secondary changes to compensate for such damage.

Functional connectivity impairments are also observed in individuals with IA. A recent study showed that subjects with IGD exhibited increased functional connectivity in the bilateral cerebellum posterior lobe and middle temporal gyrus compared with the control group [50]. The bilateral inferior parietal lobe and right inferior temporal gyrus exhibited decreased connectivity. Another study reported that adolescents with IA showed reduced functional connectivity mainly involving cortico-subcortical circuits, and bilateral putamen was the most extensively involved subcortical brain region [51]. These results suggest that IA is associated with a widespread and significant decrease of functional connectivity spanning a distributed network.

It has been reported that impulsivity is associated with IA [52]. The ability to suppress a planned motor response is usually investigated using stop-signal or go/no-go paradigms [53]. A recent study evaluated response inhibition and error processing in subjects with IGD [54]. All subjects performed event-related go/no-go task under fMRI and completed questionnaires related to IA and impulsivity. The IGD group got a higher score for impulsivity and exhibited higher brain activation when processing response inhibition over the left OFC and bilateral caudate nucleus than controls. The OFC has been associated with response inhibition [37, 55]. Therefore, these results support the fact that the fronto-striatal network involved response inhibition. A similar study examined the neural correlations of response inhibition in males with IA using an event-related fMRI Stroop color-word task [56]. The IA group demonstrated significantly greater “Stroop effect”-related activity in the ACC and PCC compared with healthy controls. The ACC has been shown to be involved in conflict monitoring and cognitive control [57, 58]. The greater ACC recruitment during Stroop color-word task may reflect diminished “cognitive efficiency” in the IA group. The PCC is a central part of the default mode network and has implicated in attentional processes [59]. The greater activation in the PCC could indicate incomplete disengagement of the default mode network resulting in failure to optimize task related attentional resources in the IA group. These results suggest that individuals with IA exhibit diminished efficiency of response-inhibition processes.

Regional homogeneity is a widely used method in fMRI studies that measures the functional coherence of a given voxel with its nearest neighbors, and it can be used to evaluate resting-state brain activities based on the hypothesis that spatially neighboring voxels should have similar temporal patterns [60]. IGD subjects showed a significant increase in regional homogeneity in the inferior parietal lobe, left posterior cerebellum, and left middle frontal gyrus and decreased regional homogeneity in temporal, occipital, and parietal brain regions compared with healthy controls [61]. The results suggest that long-time online game playing enhanced brain synchronization in sensory-motor coordination related brain regions and decreased excitability in visual and auditory related brain regions.

Several studies investigated brain areas associated with cue-induced gaming urges [62–65]. The participants were presented with gaming pictures while undergoing fMRI. These studies showed increased signal activity in distributed brain areas (e.g., dorsolateral PFC, inferior parietal lobe, ACC, parahippocampal gyrus, OFC, and PCC) in addicted group compared with control group. The activated brain regions were positively correlated with self-reported gaming urges. Abnormalities in these brain regions have been implicated in addiction by numerous studies and may be associated with dysfunctions in cognitive control, craving, goal-directed behavior, and working memory in IGD subjects [66].

An interesting study compared IGD subjects with subjects in remission from IGD and controls in cue-induced craving to play online games [67]. Bilateral dorsolateral PFC, precuneus, left parahippocampal gyrus, PCC, and right ACC were activated in response to gaming cues in the IGD group compared with the control group. These activated brain regions represent brain circuit corresponding to the mechanism of substance addiction [38, 39, 59]. Furthermore, the remission group showed reduced activation over right dorsolateral PFC and left parahippocampal gyrus than did the IGD group. Thus, the authors suggest that the two areas would be candidate markers for current addiction to online gaming.

MRI has also been used to assess therapeutic effects of specific pharmacological treatment on IA. Bupropion is a norepinephrine/dopamine reuptake inhibitor, which has been used in the treatment of patients with substance abuse. A study explored the possible effectiveness of bupropion, assessed brain activity in response to game cues using fMRI [68]. IGD showed higher activation in the left occipital lobe, left dorsolateral PFC, and left parahippocampal gyrus than controls. After 6 weeks of bupropion treatment, the craving and the total time spent gaming were lower. The cue-induced brain activity in dorsolateral PFC was also decreased, which indicated that bupropion was effective. As previously mentioned, IGD individuals in remission showed reduced activation over right dorsolateral PFC and left parahippocampal gyrus [67]. Therefore, molecular imaging has the potential to help clinicians determine the most appropriate treatment for individual patients and monitor their progress toward recovery.

## 3. Nuclear Imaging Findings

Nuclear imaging approaches, which include SPECT and PET, have the advantages of high intrinsic sensitivity, unlimited depth penetration, and a broad range of clinically available molecular imaging agents [70]. SPECT and PET provide insight into energy metabolism in vivo by quantifying glucose consumption, cerebral perfusion, and oxygen consumption. In neuroscience research, this allows the study of neural activity, as well as disease processes, based on the brain's metabolism and function [71]. PET has the additional advantages of providing higher spatial resolution than SPECT. In addition to measurements of cerebral metabolism, PET and SPECT also enable more specific analyses of neurotransmitter binding site density through the use of specific neuroreceptor radiotracers [72].

### 3.1. PET Imaging of Brain Metabolic Changes

Using ^18^F-fluoro-deoxyglucose (^18^F-FDG) PET imaging, a study investigated the differences of cerebral glucose metabolism at resting state between young individuals with IGD and those with normal use [73]. Imaging results indicated that IGD had increased glucose metabolism in the right middle OFC, left caudate nucleus, and right insula and decreased metabolism in the bilateral postcentral gyrus, left precentral gyrus, and bilateral occipital regions compared with normal users. The results suggest that IGD may be associated with neurobiological abnormality in the OFC, striatum, and sensory regions, which are implicated in impulse control, reward processing, and somatic representation of previous experiences.

### 3.2. Nuclear Imaging of Neuroreceptor Abnormalities

Emerging evidence has shown that the dopaminergic system is involved in drug addiction [74, 75]. A pilot study conducted by Koepp et al. used ^11^C-labelled raclopride and PET scans to investigate endogenous dopamine release in the human striatum during a video game [76]. Binding of the radioligand ^11^C-raclopride to dopamine D2 receptors is sensitive to levels of endogenous dopamine, which can be detected as changes in binding potential of the radioligand. The authors reported that binding of ^11^C-raclopride to dopamine receptors in the striatum was significantly reduced during the video game compared with baseline levels of binding, which suggested increased release and binding of dopamine to its receptors. Moreover, they showed that there is a significant correlation between performance level during the task and reduced ^11^C-raclopride binding potential in the striatum. Similar results have been reported in people with IA [77]. Individuals with IA had reduced dopamine D2 receptor availability in the striatum compared with controls. Furthermore, there was a negative correlation of dopamine receptor availability with IA severity. These findings are supportive of Han et al. who investigated the genetic polymorphisms of the dopaminergic system in a group of excessive Internet game players [78]. They reported that individuals with increased genetic polymorphisms in genes coding for the dopamine D2 receptor and dopamine degradation enzyme were more susceptible to excessive Internet gaming compared with age-matched controls.

Dopamine transporter is a plasma membrane protein that actively translocates released dopamine from the extracellular space into the presynaptic neurons [79]. Altered dopamine transporter concentration in the striatum following chronic substance administration has been reported previously [80, 81]. Using SPECT with the radiotracer ^99^mTc-TRODAT-1, our group investigated striatal dopamine transporter density in IA subjects to identify potential presynaptic abnormalities [82]. We showed that dopamine transporter expression level was significantly decreased and the volume, weight, and ^99^mTc-TRODAT-1 uptake ratio of corpus striatum were greatly reduced in individuals with IA compared with controls. Taken together, these results suggest that IA is associated with dysfunction of the brain dopaminergic systems.

**Figure 2:**
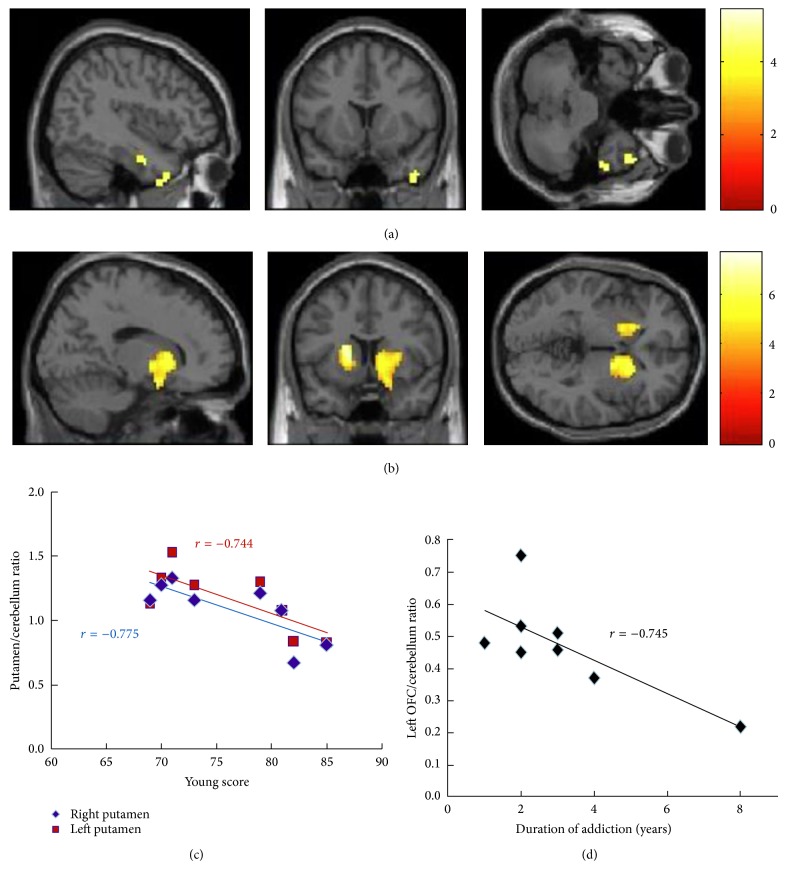
^11^C-NMSP PET imaging of dopamine D2 receptor availability in IGD subjects. (a) In the resting state, low level of ^11^C-NMSP binding was found in the right inferior temporal gyrus in the IGD subjects compared to controls (yellow color) (*P* < 0.001 uncorrected, *k* = 100). (b) In the game task state, ^11^C-NMSP binding in the putamen was significantly lower in the IGD group than the control group, especially in the right side (yellow color) (*P* < 0.001 uncorrected, *k* = 100). (c) Both right (*P* = 0.024, *r* = −0.775) and left putamen ^11^C-NMSP binding potential (*P* = 0.034, *r* = −0.744) correlated negatively with the Young score in the IGD subjects. (d) The left OFC to the cerebellum ratio of ^11^C-NMSP binding correlated negatively with the duration of internet overuse (*P* = 0.034, *r* = −0.745) [69].

In a more in-depth study, our group investigated both dopamine D2 receptor and glucose metabolism in the same individuals using PET with ^11^C-N-methylspiperone (^11^C-NMSP) and ^18^F-FDG, in both states of resting and internet gaming task [69]. A significant decrease in glucose metabolism was observed in the prefrontal, temporal, and limbic systems in IGD subjects. In the resting state, low level of ^11^C-NMSP binding was found in the right inferior temporal gyrus in the IGD subjects compared to normal controls (Figure 2(a)). After Internet gaming task, ^11^C-NMSP binding potential in the striatum was significantly lower in IGD subjects compared with controls, indicating reduced level of dopamine D2 receptor (Figure 2(b)). Dysregulation of dopamine D2 receptor was correlated to years of Internet overuse (Figure 2(d)). Importantly, in IGD subjects, low level of dopamine D2 receptor in the striatum was correlated with decreased glucose metabolism in the OFC. These results suggest that dopamine D2 receptor mediated dysregulation of the OFC could underlie a mechanism for loss of control and compulsive behavior in IGD subjects.

From these results, it appears that IA shares similar neurobiological mechanisms with drug addiction. However, there is evidence indicates that there are substantial differences in the neurobiological mechanisms of different drug addiction [83]. In a perspective article, Badiani et al. provided evidence that opiate addiction and psychostimulant addiction are behaviorally and neurobiologically distinct, and these differences might also apply to other addictions [83]. Thus, understanding the neurobiological mechanisms underlying IA is essential for the development of specific and effective treatment approaches.

## 4. Conclusions and Future Perspectives

Emerging evidence has shown that changes in brain structure and activity related to IA are relevant to brain regions involved in reward, motivation, and memory, as well as cognitive control. Molecular and functional imaging techniques have been increasingly applied to IA research, contributing significantly to our understanding of the neurobiological mechanism. Most of the previous literatures have studied IA individuals only under resting state, verified structural and functional abnormalities in the OFC, dorsolateral PFC, ACC, and PCC. Those regions may play crucial roles in salience attribution, inhibitory control, and decision making. So far, only one PET study with ^11^C-NMSP and ^18^F-FDG was conducted under both resting and Internet gaming task states in the same individuals (either with IGD or not) and found that dopamine D2 receptor mediated dysregulation of the OFC could underlie a mechanism for loss of control and compulsive behavior in IGD subjects.

As IA has become a serious problem worldwide, a need for effective treatment is becoming increasingly urgent. Both psychological and pharmacological treatment approaches have been applied to treat IA. Several drugs have shown to be promising in treating IA, such as antidepressants, antipsychotics, and opioid receptor antagonists [84]. Cognitive-behavioral therapy has been applied to treat substance abuse [85]. Since IA appears to share similar mechanism with substance abuse, cognitive-behavioral therapy has also been verified to be effective in treating IA [86]. Further research using various specific radiotracers to target other neurotransmitter systems affected by IA will provide a more complete picture of the neurobiological mechanism that underlie IA. Moreover, specific radiotracers could be used to assess therapeutic effects of specific pharmacological treatment, for example, using ^11^C-carfentanil to study the mu-opioid receptor availability and predict treatment outcomes of opioid receptor antagonists and help clinicians determine the most appropriate treatment for individual patients.
